# Cholera, and Its Relation to Pregnancy and Child-Birth

**Published:** 1871-09

**Authors:** Carl Proegler

**Affiliations:** Late Surgeon German Army, Aurora, Ill.


ARTICLE VI.—*Cholera, and its Relation to Pregnancy and Child-Birth.* By CARL PROEGLER, M.D., late Surgeon German Army, Aurora, Ill.

As we may expect the approach of cholera again, it will not be without interest to a good many physicians, who have not had occasion, perhaps, to study that fatal disease, and I will endeavor to give some of my experience. It was during an epidemic in the southern part of Germany (Munich), that I had occasion to make the following observations:

Pregnant women may get cholera in any stage of pregnancy, as the following statement will show. These patients were admitted up to a certain day. (In speaking of month, I mean lunar month.)

Of 36 pregnant women admitted, 5 were in their 10th month; 4 in their 9th; 3 in their 8th; 4 in their 7th; 2 in their 6th; 3 in their 5th; 8 in their 4th; 3 in their 3rd; and 4 in their 2nd.

There is no month with a certain immunity, and no certain predisposition; and the question whether cholera in pregnancy is more fatal than otherwise, will be rather difficult to decide.

Certainly any disease complicated with pregnancy is itself more grave, and certainly we may think that a non-pregnant woman without cholera will sooner get well than a pregnant woman with it. It is certainly very hard to decide at the first glance what grade of infection a cholera patient has; and I remember a good many patients brought to the hospital with nothing else than slight diarrhœa, who would with the utmost care die in collapse. As in other cases, so with patients, pregnant and with cholera, the infection is the only thing decisive for the duration and cure. We lost 21 out of 36—mostly extreme cases.

Cholera in pregnancy is almost similar to that without it. Only the pains experienced by those affected with asphyxia (collapse) along the region of the kidneys seem to be, with pregnant women, more severe. It is rather difficult to decide about the causes of these pains, whether they are seated in the kidney itself, or whether they are only sympathetic pain arising from the rectum. It may be possible that with pregnant women, pains occur in the state of asphyxia by painful contraction of the uterus, pains across the kidney may simultaneously arise.

Three times I have seen death ensuing with protracted asphyxia; seven pregnant women died with asphyxia of a shorter duration, and eight in the cholera typhoid (collapse). There is nothing abnormal in these deaths compared with cases of non-complicated cholera. Cramps which can be noticed with other non-complicated cases occurred rather frequently with pregnant women, but were probably due to uræmia.

We have seen that pregnancy does not modify the causation of cholera; let us now see how much pregnancy and child-birth will be influenced by cholera.

My diary shows, that cholera not very seldom interrupts pregnancy. Of the twenty-one deaths by cholera, we had eight which died before death of the fœtus.

In the eight cases nature accomplished premature delivery. Of the remaining fifteen, five aborted. Delivery especially comes on more easy in the later months than earlier. Of all the women who were delivered, there were only two in the third month of pregnancy; all the rest were in the seventh, eighth, ninth and tenth lunar months.

Of the other women not delivered and who died, only four had passed the sixth month, and they became in so short a time asphyxiated, died so soon after cholera set in, that nature, if I may say so, had not time to commence the act of delivery. Of all those who did not abort and lived through, none had passed the tenth fifth months.

In accordance with Bouchat and Deasche (see the latter, Epidemic Cholera, page 295,) it was impossible to make exact observations, because we would get the cases where no fœtal life could be traced. I could not discover, like Deasche, first more frequent pains and afterwards gradually wearing away; it was apparent that pains were retarded and pains themselves not strong enough.

Pains commence in pauses of thirty minutes to one hour, lasting only a few seconds, but are of little use, because the woman will hardly, as the common saying is, "bear down"; delivery is, in consequence, delayed more so than generally with multiparas, where there is no mechanical impediment. There are few exceptions, and I have seen very good pains in two cases. After delivery the uterus contracts well. Only in one case have I seen slight hemorrhage on account of atony of the uterus, which was easily controlled by cold applications and Sec. cornut. in 10 grain doses.

In women who have been delivered by sectio cæsarea, I noticed that the contractility of the uterus after death was not in accordance with the spinal muscles (Wirbelmuskeln).

I noticed on those patients post-mortal contraction of the muscles, especially at the extremities and also at the mm. pectorales, but the uterus, after delivery of child and placenta, was flabby-like. I had no means of using electricity, else I would have tried to compare the muscles of the uterus with the other muscles of the body. The involution of the uterus in the puerperium is a good one, if not complicated with typhoid. Three times I noticed diphtheritis vaginæ resulting in death; only one came to dissection, and in this it was found that diphtheritis was involving the uterus.

We used the same mode of treatment with pregnant women as with other patients. The patients were rubbed with ice, cold applications to the abdomen, and wine and soda water internally; but in slight cases, for preventing abortus, we did not use cold applications as freely.

There are certain indications where an active course on the part of the accoucheur should be pursued. Pregnancy itself, and especially complicated with cholera, is more dangerous than occurring alone, therefore I think the indications are to deliver as speedily as possible. As soon as the head presents and the forceps can be applied, the delivery ought to be made as quick as possible, even if the os is not quite dilated and the head not so high up. I think it safe to have resort to the forceps, especially when the pains are slow and rather weak, and perhaps the state of the mother encourages hope of recovery.

If the pelvis should be too small, craniotomy should be resorted to. I could not notice that the mortality was less with forceps, and I lost two cases where asphyxia passed off in the typhoid state, but I do think, that in such a fearful disease as cholera, every resort is admissible which promises benefit. The mortality in cholera asphyxia is about 100 per cent. if you let nature bring about delivery. And I do believe it the duty of the accoucheur, if he gets his patients soon enough and fœtal life is not extinct, to try to save both mother and child.

Premature delivery, sectio cæsarea, forced delivery, turning, etc., would be the indication to save mother and child.

For premature delivery the cholera attack does not leave time—the fœtus dies sooner than you can deliver; to try the sectio cæsarea with a pregnant woman with cholera asphyxia, would bring the life of the mother in jeopardy for the questionable life of the fœtus—mortality itself is in cæsarean section almost as fatal as in cholera; forced delivery (forceps) is hardly any better, but should be tried, because in cholera there is everything to gain and nothing to lose; the same I can say with turning.

As noticed before, the prognosis for the fœtus in pregnant cholera women is very unsatisfactory. I have not seen a case where, after asphyxia set in, the fœtus would live. The prognosis is a little more favorable in cases where the mother has gone through a hard attack of cholera without asphyxia.

The nearer the development of fœtal life the less are the chances of its recovery; in the seventh lunar month every fœtus may be considered lost, if delivery has to be accomplished by nature.

Pains invariably ceaše as soon as purging sets in; but, on the other hand, I found the fœtus yet alive in a beginning state of collapse. The changes of the dead fœtus in utero are the same—it macerates and decomposes. It is very hard to determine whether the liquor amni will diminish during an attack of cholera. As far as I can remember, the quantity was the same in a case of cæsarean section, even greater. The question as to what destroys the fœtus will be equally difficult to answer.

I dissected almost every fœtus which was not decomposed, or saw it dissected. But one post-mortem resembles the other. I shall give here the post-mortem, and each one may form his own opinion. I confess that I first took what I saw for cholera, but now I have changed my opinion, because our greatest pathologists have seen the same, and in children where there was no cholera.

Opening the abdomen you will find the ileum rose colored, especially the upper part, the lower part with a greenish hue; the same with the duodenum, which color was still greener. About three and a half inches of the ileum were filled with a colorless, whitish, dead epithelium. The lower part of the ileum with same pale green tenacious mass, getting darker towards the duodenum. In cutting the upper part of the ileum, a watery fluid escaped with some white flocks; the membrane on the upper part rose-colored, the vessels strongly injected; the membrane of the stomach rose-colored, with some streaky ecchymoses. The contents of the stomach contained opaque-colored fluid, intermixed with white stripes.

Strong subpericardial ecchymoses of the heart, especially in the neighborhood of the coronary nerve. Thymus greatly studded with ecchymoses.

Subpleural hemorrhage on the lungs; lungs without air, and the lower parts strongly filled with blood.

In the bronchii nothing resembling vernix caseosa; liver extremely pale and bloodless; spleen, nothing remarkable; kidneys, a strong line of demarcation between the cortical and medullar substance; the first a little yellow, the latter filled with blood of a dark blue red color.

Brain very soft, filled with blood.

Bladder—a small quantity of pale yellow urine.

It cannot be denied that the result is very similar to what we see in cholera dead in the adult, and it explains the view Güterbock took in saying the fœtus dies with cholera.

In conjunction with my article, I will give below a few experiments made with animals, to decide the following questions:

1. Are the dejections of cholera-sick adults poisonous for animals?

2. Can we see the same symptoms as Asiatic cholera in animals?

3. Are the same pathological changes to be seen in animals which are characteristic of cholera?

I used the injections either by rectum or hypodermically, and took the rice-water stools of patients who died in the state of asphyxia, either fresh or about three days old. Some were filtered, and some not.

I injected in a large rabbit about 72 centigrammes, or about 12 grains, of fresh filtered rice-water stool between the skin of the back. The animal seemed to be very dry, and drank about thirty centigrammes, or five grains, of the same fluid. But very soon it refused to take any food. With increasing drowsiness death ensued after eighteen hours, without having had diarrhœa.

Sectio cadaveris showed the ileum very red, the vessels filled with blood, some glands of Peyer, of the size of a bean, some smaller, protruding more than an inch from the mucous membrane and very strongly injected, even showing small hemorrhagias in the follicles. The vessels leading to the glands of Peyer were strongly filled with blood. Solitary follicles not abnormal. The contents of the ileum consisted of a slimy mass of a whitish color; the contents of the duodenum contained soft feces. The heart did not show anything abnormal, neither the lungs; ecchymoses, either subpericardial or subpleural, not present. In the kidneys there was microscopically nothing of pathological interest, but microscopically the epithelium of the tubi uriniferi was found partly fatty degenerated, partly broken down tissue. The bladder was filled with pale urine.

Another rabbit was injected with 7 grains=42 centigrammes of good rice-water stool. The animal was for the next twenty-four hours perfectly well. Twenty-six hours afterwards I injected 70 centigrammes=12 grains in different parts under the skin. The animal died twelve hours afterwards.

Dissection did not reveal anything extra. The contents of the duodenum were fecal; ileum contained bile-colored masses. The stomach was filled with food. The mucous membrane of the ileum was pale, glands perfectly normal, heart, lungs and kidneys perfectly sound.

Another rabbit, who fasted a day, took 60 centigrammes of filtered rice-water stool by the stomach. The animal remained perfectly well. The same fluid injected subcutaneously the next day killed it in about twenty-four hours. Nothing pathological to be found.

I injected under the skin of a dog, 75 centigrammes of one and a half days not filtered rice-water stool. The dog, whom I kept round the house, and who was always pretty lively, became quite changed from the moment of the hypodermic injection.

He did not take any food, was apathetic to all persons, and died in a perfectly comatose state. Before death he vomited; no diarrhœa.

Post mortem three hours afterwards, revealed great rigor mortis, the muscles normally colored, but a little dry; heart, lungs and kidneys perfectly sound. The stomach contained quite a large quantity of whitish froth. The ileum contained bile-colored mucus; the duodenum, fecal matter. The mucous membrane of the whole intestine pale; no glandular enlargement. The color of the blood in the greater veins with this and the other animals very dark. The right ventricle contained always dark coagulated blood.

I was inclined to take the first case for cholera, but after due reflection, I changed my mind, because the glands of Peyer are sometimes in normal relations with rabbits very great, just so with the fatty degeneration of the kidneys. I conclude, therefore—

1. Dejection of cholera will act as poison on animals subcutaneously injected, either fresh, old, filtered or not filtered.

2. The same amount of stools taken by the stomach acts not poisonous, even without a sign of sickness.

3. The death of the animals takes place without our being able to prove the cause of death.

4. The animals do not die with the symptoms of cholera, and to use rather a vague expression, they die with pyæmia.

